# Supplementary material for: Assessing the Effect of Silicon Supply on Root Sulfur Uptake in S-Fed and S-Deprived Brassica napus L
Source: Plants (Basel). 2022 Jun 18;11(12):1606. doi: 10.3390/plants11121606 (PMC9227570; doi:10.3390/plants11121606)
Supplement: Supplementary file 1 [file plants-11-01606-s001.zip › plants-1758297-supplementary.pdf]

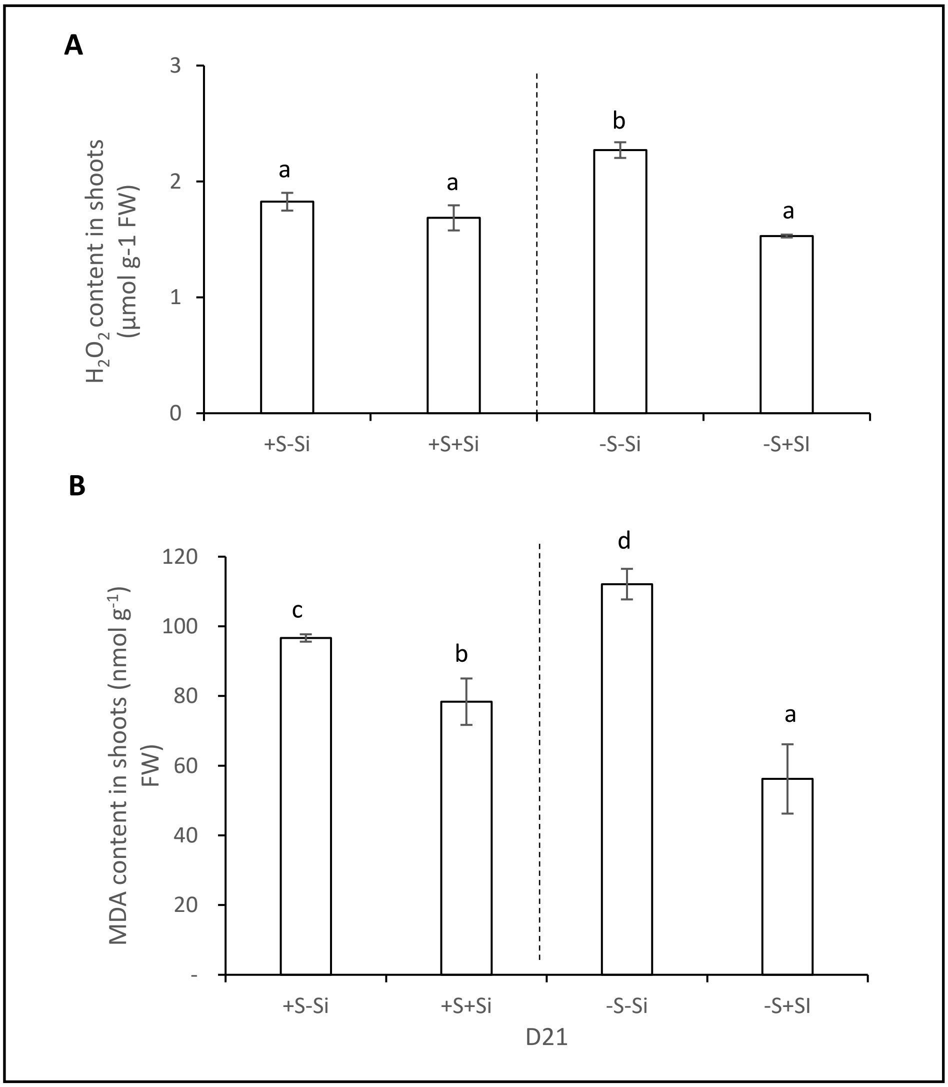


Supplemental data S1: Hydrogen peroxide ($H_2O_2$) and malondialdehyde (MDA) contents in shoots of *Brassica napus* L cultivated in hydroponic conditions for 21 days (D21) with (+S; 500 µM) or without sulfur (–S; 0 µM) concentrations and with (+Si; 1.7 mM) or without silicon (-Si) supply. Data are means ± SE (for n = 4). Different lowercase letters indicate significant difference in the indices ($p < 0.05$). Hydrogen peroxide ($H_2O_2$) and malondialdehyde (MDA) contents were determined according to methods previously described by Haddad et *al.* [28].
